# Supplementary material for: MetaQTL: a package of new computational methods for the meta-analysis of QTL mapping experiments
Source: BMC Bioinformatics. 2007 Feb 8;8:49. doi: 10.1186/1471-2105-8-49 (PMC1808479; doi:10.1186/1471-2105-8-49)
Supplement: Additional File 7 — MetaQTL Package : jar file and tutorial. This Zip archive contains both the MetaQTL JAR file and the files of the tutorial. [file 1471-2105-8-49-S7.zip › org.inra.metaqtl/doc/org/metaqtl/numrec/class-use/NumericalUtilities.Function.html]

Uses of Interface org.metaqtl.numrec.NumericalUtilities.Function


|  |  |  |
| --- | --- | --- |
| **Overview** \| **Package** \| **Class** \| **Use** \| **Tree** \| **Deprecated** \| **Index** \| **Help** | |  |
| PREV   NEXT | **FRAMES**    **NO FRAMES**     **All Classes** |  |


---


## **Uses of Interface org.metaqtl.numrec.NumericalUtilities.Function**

| Packages that use NumericalUtilities.Function | |
| --- | --- |
| **org.metaqtl.numrec** |  |

Uses of NumericalUtilities.Function in org.metaqtl.numrec

| Methods in org.metaqtl.numrec with parameters of type NumericalUtilities.Function | |
| --- | --- |
| `static double` | `NumericalUtilities.dfridr(NumericalUtilities.Function f, double x, double h)`             Returns the derivative of a function func at a point x by Ridders' method of polynomial extrapolation. |

---


|  |  |  |
| --- | --- | --- |
| **Overview** \| **Package** \| **Class** \| **Use** \| **Tree** \| **Deprecated** \| **Index** \| **Help** | |  |
| PREV   NEXT | **FRAMES**    **NO FRAMES**     **All Classes** |  |


---
